# What is the impact of post-COVID-19 syndrome on health-related quality of life and associated factors: a cross-sectional analysis

**DOI:** 10.1186/s12955-023-02107-z

**Published:** 2023-03-22

**Authors:** Ilaria Mastrorosa, Giulia Del Duca, Carmela Pinnetti, Patrizia Lorenzini, Alessandra Vergori, Anna Clelia Brita, Marta Camici, Valentina Mazzotta, Francesco Baldini, Pierangelo Chinello, Paola Mencarini, Maria Letizia Giancola, Amina Abdeddaim, Enrico Girardi, Francesco Vaia, Andrea Antinori

**Affiliations:** 1 Clinical Department of Infectious Diseases and Research, National Institute for Infectious Diseases Lazzaro Spallanzani IRCCS, Rome, Italy; 2 National Center for Disease Prevention and Health Promotion, National Institute of Health, Rome, Italy; 3 Psychology Service, Clinical Department of Infectious Diseases and Research, National Institute for Infectious Diseases Lazzaro Spallanzani IRCCS, Rome, Italy; 4 Scientific Direction, National Institute for Infectious Diseases Lazzaro Spallanzani IRCCS, Rome, Italy; 5 General Direction, National Institute for Infectious Diseases Lazzaro Spallanzani IRCCS, Rome, Italy

**Keywords:** Post-COVID-19 syndrome, Post-acute sequelae of COVID-19 infection (PASC), Post-COVID-19 condition, Long COVID, Health-related quality of life, The Short-Form 36-item questionnaire, Physical health, Mental health, Neuropsychiatric symptoms, Sleep disorders

## Abstract

**Background:**

After the acute phase, symptoms or sequelae related to post-COVID-19 syndrome may persist for months. In a population of patients, previously hospitalized and not, followed up to 12 months after the acute infection, we aim to assess whether and to what extent post-COVID-19 syndrome may have an impact on health-related quality of life (HRQoL) and to investigate influencing factors.

**Methods:**

We present the cross-sectional analysis of a prospective study, including patients referred to the post-COVID-19 service. Questionnaires and scales administered at 3, 6, 12 months were: Short-Form 36-item questionnaire (SF-36); Visual Analogue Scale of the EQ5D (EQ-VAS); in a subgroup, Beck Anxiety Inventory (BAI), Beck Depression Inventory (BDI-II) and Pittsburgh Sleep Quality Index (PSQI). Linear regression models were fitted to identify factors associated with HRQoL.

**Results:**

We considered the first assessment of each participant (*n* = 572). The mean scores in SF-36 and in EQ-VAS were significantly lower than the Italian normative values and remained stable over time, except the mental components score (MCS) of the SF-36 and EQ-VAS which resulted in lower ratings at the last observations. Female gender, presence of comorbidities, and corticosteroids treatment during acute COVID-19, were associated with lower scores in SF-36 and EQ-VAS; patients previously hospitalized (54%) reported higher MCS. Alterations in BAI, BDI-II, and PSQI (*n* = 265)were associated with lower ratings in SF-36 and EQ-VAS.

**Conclusions:**

This study provides evidence of a significantly bad perception of health status among persons with post-COVID-19 syndrome, associated with female gender and, indirectly, with disease severity. In case of anxious-depressive symptoms and sleep disorders, a worse HRQoL was also reported. A systematic monitoring of these aspects is recommended to properly manage the post-COVID-19 period.

## Introduction

SARS-CoV-2 infection, after the end of the acute phase, can result in heterogeneous clinical manifestations that preclude a full return to the previous state of health [1, 2]. Quality of life is a multidimensional concept that refers to an “individuals’ perception of their position in life in the context of the culture and value systems in which they live and in relation to their goals, expectations, and standards” and is influenced by both, physical and mental health *status* of a person [3]. The measure of health perception in the population is crucial to assess the benefit of health care interventions and to target services. A comprehensive assessment of quality of life require instruments able to capture subjectivity and multidimensionality. To be of practical use, the measure must be brief, easy to use and comprehensive. The Short-Form 36-item questionnaire (SF-36) and the Euro-Qol-5 Dimension (EQ-5D) include these features, in fact they are widely used to assess multi-dimensional domains of health and well-being of different target populations [4]. “Long COVID” is commonly used to describe signs and symptoms that continue or develop after acute COVID-19, both in *ongoing symptomatic COVID-19* (from 4 to 12 weeks after the acute phase) and in *post-COVID-19 syndrome* (for more than 12 weeks and not explained by an alternative diagnosis) [3]. It has been estimated that it may occur in up to 25% of patients [1, 5]. The post COVID-19 syndrome may have a negative impact on health-related quality of life (HRQoL), but the majority of the studies uses not homogeneous generic HRQoL assessment tools, and only few studies include a systematic assessment through valid instruments [2, 4, 6]. Furthermore, little is known about long-term sequelae, as the available reports present short observation periods limited to the first 6 months after acute infection, few up to 12 months [2, 7]. In addition, almost all the published analyses are focused on previously hospitalized patients [2, 6, 7], rather than non-hospitalized patients with asymptomatic or pauci-symptomatic forms of COVID-19, who represent the majority of the population. Finally, understanding the burden of this condition, and who is at greatest risk of lower HRQoL due to COVID-19 long-term complications, may help to target preventive strategies and provide support for rehabilitation and targeted interventions [7–10].

Therefore, we planned to assess, through validated instruments, whether and to what extent post-COVID-19 syndrome may have an impact on self-reported HRQoL and perceptions of physical and mental health *status*, in a population including patients with and without previous hospitalization, followed up to 12 months after acute infection, enrolled in the NEUROCOVID study; in addition, we investigated the factors influencing different perceptions of HRQoL and health *status* in order to identify those patients deserving of tailored interventions.

## Methods

### Study design and study population

NEUROCOVID is an ongoing prospective and monocentric study conducted at the National Institute for Infectious Diseases “L. Spallanzani” IRCCS in Rome, Italy, enrolling patients during the acute phase of COVID-19 and/or during the post-COVID-19 period. The study protocol (NeuroCovid Study, version 2.0, January 08, 2021), approved by the Ethical Committee of the Institute (approval number 265/2021), included four main sub-studies, two of which aimed (1) to assess patients’ neuropsychological profile and (2) to evaluate HRQoL self-reported by the patients. Patients were asked to adhere to one or more specific sub-studies, driven by clinical decision. All the patients with 18 years or older and with documented SARS-CoV-2 infection, ongoing or previous, were considered eligible after having signed a specific informed. Patients with cerebral focal pathologies, major depression in progress, presence of psychosis and other serious psychiatric pathologies, current drug use, opioid treatment, abuse of psychiatric drugs, visual impairment, motor deficit of the dominant hand, mental delay, lack of command of the Italian language and cultural disadvantage were excluded from the study.

Here, we present a cross-sectional analysis including patients referred to our post-COVID-19 outpatient service, with and without prior hospitalization, by considering only their first access, from June 2020 to October 2021, evaluated 3 months after the acute infection and, according to clinical decision, at 6 and 12 months, approximately. Each participant agreed to be enrolled in the second sub-study (HRQoL evaluation), a subgroup in the first one (neuropsychological assessment). Demographic, pharmacological and clinical data, including information on comorbidities (cardiologic, respiratory and neurologic diseases, active malignancies and diabetes), were collected anonymously into an Electronic Case Report Forms (eCRF); subjects were identified by numeric codes only, password protected.

### Questionnaires

The psychological instruments used in this study were chosen based on their psychometric properties and ability to measure self-perception of health *status* and HRQoL [11, 12]. This assessment was carried out through two specific tools SF-36 and EQ-5D, and patients were asked to fill them at each visit. The validated Italian version of both instruments, SF-36 [13] and EQ-5D (available at the EuroQol website http://www.euroqol.org), was administered and Italian normative values reported in earlier studies were used as comparison [13–15].

The SF-36 [13, 16] is a self-administered questionnaire containing 36 items which takes about five/ten minutes to be completed; it may be applied to people having many different types of treatment or conditions and in all the different states of health. It is one of the most widely employed generic measures of HRQoL, used to evaluate individual patients, to estimate the cost-effectiveness of a treatment and to monitor and compare the disease burden; in fact, it has been shown to discriminate between subjects with different chronic conditions and between subjects with different severity levels of the same disease [13, 17]. In addition, the Italian version of the SF-36 has demonstrated a high degree of reliability [13]. The SF-36 questionnaire measures mental and physical health by considering eight multi items dimensions, covering functional *status*, well-being, and overall evaluation of health: items are claimed to detect positive as well as negative states of health. In six of the eight dimensions, patients are asked to rate their responses on three- or six-point scales (box). For each dimension, item scores are coded, summed, and transformed on to a scale from 0 (worst health) to 100 (best health). The SF-36 comprises eight health scales: physical role functioning (PF, 10 items), role limitations–physical (RP, 4 items), bodily pain (BP, 2 items), general health perceptions (GH, 5 items), vitality (VT, 4 items), social role functioning (SF, 2 items), emotional role functioning (RE, 3 items), and mental health (MH, 5 items). Two core dimensions of health, physical (PH) and mental (MH), can be derived from these eight scales in order to obtain a mental components score (MCS) and a physical components score (PCS). The exact balance between the physical and the mental components and their contributions to the HRQoL is unknown, thus we did not calculate any global score of HRQoL such as the “SF-36 Total/Global/Overall Score”, a measure with poor validity, which is not supported by the SF-36 developers [18].

The EQ-5D [19, 20] was built with the aim of becoming a generic and extremely flexible tool for measuring the self-perception of quality of life, short and easy to use in self-administration. Although recently an expanded five-level version of the EQ-5D instrument (EQ-5D-5L) has become available and was translated for use across countries, the original three-level version of the EQ-5D (EQ-5D-3L), here referred to as EQ-5D, was used for our study. The EQ-5D is made up of two separate sections. The first section asks for a subjective evaluation of five dimensions of health (mobility, self-care, daily activities, pain/ discomfort and anxiety/depression) on three severity level (1. no problems, 2. some problems, 3. extreme limitation). The aggregation of the answers represents the respondent's state of health and allows to highlight the presence/absence of any problems and their intensity. The second section of the EQ-5D includes a self-rating of “your health today” using a visual analogue scale (VAS) graphically represented by a graduated scale ranging from 0 (the worst possible state of health) to 100 (the best possible health state) on which the interview indicates their perceived level of health. For the present study, we considered only the VAS score of the EQ-5D (EQ-VAS) with showed a moderate level of reliability in our country [21].

### Neuropsychiatric symptom assessment

In a subgroup of patients enrolled in the sub-study consisting of a complete neuropsychological assessment, the Beck Anxiety Inventory (BAI), the Beck Depression Inventory (BDI II) and the Pittsburgh Sleep Quality Index (PSQI) were administered in order to assess the presence of anxiety and depressive symptoms and sleep disorders, respectively.

The BAI is a self-report tool that allows to assess the severity of anxiety symptoms in adults, through a questionnaire of 21 items (descriptions of symptoms of somatic, subjective or phobia-related anxiety), to be evaluated on a four-point scale (from 0 to 3). Scores > 85% indicates the presence of pathological symptoms (85–90 condition of anxiety bordering on pathological aspects; 91–95 anxiety condition that causes discomfort and difficulty for the subject; > 95 particularly alarmed reaction index for the subject) [22, 23]. The BDI-II is a self-report tool that allows to assess the presence and intensity of symptoms correlated to depression. This test of 21 items returns a total score and two other scores relating to somatic-affective area (which concerns the somatic-affective manifestations of depression such as loss of interest, loss of energy, changes in sleep and appetite, shaking and crying) and to cognitive area (which concerns cognitive manifestations such as pessimism, guilt, self-criticism). Scores > 85% indicates the presence of pathological symptoms (85–90 condition of dysphoria bordering on pathological aspects; 91–95 condition of dysphoria that causes discomfort and difficulty for the subject; > 95 particularly alarmed reaction index for the subject) [24, 25]. The PSQI is a self-assessment scale consisting of 19 items, grouped into 7 composite items, evaluated on a scale from 0 to 3, which give the overall score of the PSQI, ranging from 0 to 21. These 7 composite items represent subjective quality of sleep, sleep latency, sleep duration, habitual sleep efficacy, sleep disturbances, hypnotic drug use, and disturbances during the day. If score > 5 indicates the presence of poor sleep quality [26].

The Italian version of the BAI, BDI-II and PSQI showed an overall good reliability, indicating a high degree of internal consistency [23, 25, 26].

### Statistical analyses

Descriptive characteristics were provided using medians and interquartile ranges (IQR) for continuous variables, and frequencies and percentages for categorical variables. Student’s T-test was employed to compare SF-36 and EQ-VAS mean scores to the normative values, and a linear regression was used to assess for the presence of a trend over time by month of evaluation after the acute infection (continuous measure in month). Finally, two different linear regression models were fitted. The first one was built using as dependent variable, scores in SF-36 (splitted in PCS and MCS) and in EQ-VAS, and as covariates, demographic, clinical and therapeutic variables, including distance from the acute infection. This association was studied in both uni- and multivariable analyses. In the subgroup of patients with neuropsychiatric symptoms’ evaluation, linear regression, was used in order to explore the correlation between alterations in BAI, BDI II, and PSQI, with the perception of PH and MH *status* and HRQoL. A statistically significant difference in the variables tested was indicated as *p*-value < 0.05 (two-sided). Statistical analysis was performed using STATA 15.1 software.

## Results

### Descriptive analysis

Out of a total of 914 assessments, we considered the first one of each patient (*n* = 572). The median age was 55 years (IQR 47- 62), 303 (53%) were male, 215 (38%) had at least one comorbidity, 235 (41%), 175 (31%) and 118 (21%) patients were evaluated 3 months [median 3.5 (IQR 2.9–3-9)], 6 months [5.6 (5.0–6.5)], and more than 6 months [9.6 (8.3–14.7)], after the acute infection, respectively. Patients with a previous hospitalization were 309 (54%) and the median time from acute infection was 4.8 months (IQR 3.6–7.1). General baseline patients’ characteristics and the management during the hospital admission are reported in Table 1.

**Table 1:** Patients’ characteristics at baseline

	**Characteristics of Study Population** ***n*** = 572
Demographic	
Gender	
* male, n (%)*	303 (53.0%)
* female, n (%)*	269 (47.0%)
Age, years, median (IQR)	55 (47–62)
Comorbidities	
Presence of comorbidities, *n* (%)	215 (37.6%)
Number of comorbidities	
* 1, n (%)*	152 (26.6%)
* 2, n (%)*	44 (7.7%)
≥ *3, n (%)*	19 (3.3%)
Distance from acute phase	
Months from acute phase, median (IQR)	4.8 (3.6–7.1)
* 3 months, n (%)*	235 (41.1%)
* 6 months, n (%)*	175 (30.6%)
> *6 months, n (%)*	118 (20.6%)
Previous hospitalization, n (%)	309 (54.0%)
Non-invasive ventilation, *n* (%)	104 (18.2%)
Intensive Care Unit, *n* (%)	11 (1.9%)
Treatment in the acute phase	
* corticosteroids, n (%)*	323 (56.5%)
* remdesivir, n (%)*	145 (25.4%)
* immunotherapy, n (%)*	10 (1.8%)
* heparin, n (%)*	258 (45.1%)

*IQR* InterQuartile Range, *n* number of participants

### HRQoL assessment

**Fig. 1:**
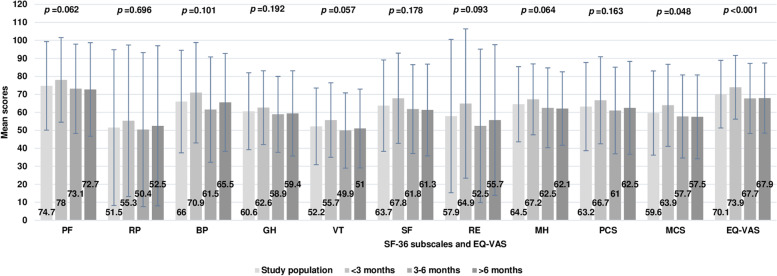
Mean scores in SF-36 subscales and in EQ-VAS, in the study population (first bar) and by month of evaluation (other bars). SF-36 the Short-Form 36-item questionnaire; EQ-VAS Visual Analogue Scale score of the EQ-5D; PF Physical Function; RP limitations due to physical health problems—Role Physical; BP Bodily Pain; GH General Health; VT vitality; SF social functioning; RE limitations due to emotional health problems—Role Emotional; MH mental health; PCS physical components score of the SF-36; MCS mental components score of the SF-36; * p-values for trend over time by month of evaluation after the acute infection, are shown

The mean scores of the two dimensions of health, PCS and MCS, investigated by the SF-36 questionnaire, were 63 (SD 25) and 60 (23), respectively. When compared to the reference population [13], the mean ratings of each subscale assessed in SF-36 were significantly lower; similarly, the mean EQ-VAS score was 70 (19) for the entire study population, lower than the Italian normative value (*p* < 0.001) [15]. Comparisons are reported in Table 2. During the period of observation, the over mentioned scores remained stable over time, with the exception of MCS and EQ-VAS which resulted in lower ratings at the last observations (Fig. 1).

**Table 2:** Mean scores in SF-36 and in EQ-VAS for study population compared to the Italian normative values

	**Study** **Population,** **mean (SD)**	**Italian Normative Population,** **mean (SD)**	p-value*
**SF-36 subscales**			
PF*, * *Physical Function*	74.7 (24.6)	84.5 (23.2)	** < ** ***0.001***
RP, *Role Physical*	51.5 (43.3)	78.2 (35.9)	** < ** ***0.001***
BP, *Bodily Pain*	66.0 (28.5)	73.7 (27.7)	** < ** ***0.001***
GH*, General Health*	60.6 (21.4)	65.2 (22.2)	** < ** ***0.001***
VT, *Vitality*	52.2 (21.3)	61.9 (20.7)	** < ** ***0.001***
SF*, * *Social Functioning*	63.7 (25.4)	77.4 (23.3)	** < ** ***0.001***
RE, *Role Emotional*	57.9 (42.6)	76.2 (37.3)	** < ** ***0.001***
MH*, Mental Health*	64.5 (20.9)	66.6 (20.9)	***0.003***
**EQ-5D**			
EQ-VAS	70.1 (18.8)	77.7 (19.1)	** < ** ***0.001***

*SD* standard deviation, *SF-36* short-form 36-item questionnaire, *EQ-VAS* visual analogue scale score of the EQ-5D

*p-values refer to student’s t-test

### Neuropsychiatric symptoms assessment

Analysis of neuropsychiatric symptoms was performed in a subgroup of a total of 256 patients and we found that scores of the BAI scale for self-reported anxiety symptoms were above the 85° percentile in 141/295 (48%) patients (85°-90° percentile anxiety bordering on pathological aspects, 91°-95° anxiety index of unease, > 95° disabling anxiety), and for the BDI-II scale for self-reported depressive symptoms, somatic-affective and cognitive symptoms respectively, were above the 85° percentile in 134/295 (45%) patients (85°-90° percentile depressive symptoms bordering on pathological aspects, 91°-95° depressive symptoms index of unease, > 95° disabling depressive symptoms). In addition, the pathological scores (≥ 5) on the PSQI scale for the self-perceived sleep quality, evaluated in the same population, were reported from 85/295 (29%) patients, highlighting alterations in sleep quality.

### Factors influencing HRQoL

The multivariable linear regression analysis showed that female gender, the presence of comorbidities and the use of corticosteroids during the acute COVID-19, were associated with lower scores in SF-36, considering PCS and MCS, and EQ-VAS, which mean a worse perception of health *status* and HRQoL; only patients with previous hospitalization reported higher scores in MCS and the use of immunotherapy during the acute phase of infection was a significant predictor of lower scores in PCS (Table 3). Finally, in the univariable linear regression model, alterations in BAI, BDI II, and PSQI were associated with lower scores in SF-36 and EQ-VAS, in the subgroup of 265 patients in whom they were evaluated (Table 4).

**Table 3:** Factors associated with higher scores in SF-36 and in EQ-VAS, by multivariable linear regression

	**PCS**				**MCS**				**EQ-VAS**			
	**beta**	**95%CI**		***p-value***	**beta**	**95%CI**		***p-value***	**beta**	**95%CI**		***p-value***
**Male ** ***vs*** ** female**	16.64	12.71	20.57	** < ** ***0.001***	15.53	11.73	19.33	** < ** ***0.001***	10.28	7.12	13.45	** < ** ***0.001***
**Age, 10 years increase**	-0.66	-2.45	1.12	*0.466*	1.38	-0.35	3.10	*0.118*	-0.32	-1.76	1.12	*0.662*
**Number of comorbidities**												
*** 1***	-7.99	-12.75	-3.24	***0.001***	-4.99	-9.59	-0.39	***0.033***	-4.79	-8.62	-0.95	***0.015***
*** 2***	-12.90	-21.03	-4.77	***0.002***	-9.91	-17.77	-2.05	***0.014***	-12.18	-18.74	-5.63	** < ** ***0.001***
** ≥ ** ***3***	-27.63	-38.76	-16.50	** < ** ***0.001***	-23.78	-34.54	-13.02	** < ** ***0.001***	-17.13	-26.10	-8.16	** < ** ***0.001***
**Months from the acute phase**	0.21	-0.06	0.48	*0.125*	0.03	-0.23	0.29	*0.817*	0.14	-0.08	0.36	*0.204*
**Previous hospitalization**	2.50	-2.55	7.55	*0.331*	5.67	0.80	10.55	***0.023***	0.62	-3.44	4.69	*0.764*
**Treatment in the acute phase**												
*** corticosteroids***	-10.18	-15.17	-5.18	** < ** ***0.001***	-6.16	-10.99	-1.33	***0.012***	-5.55	-9.58	-1.53	***0.007***
*** remdesivir***	3.89	-1.96	9.74	*0.192*	3.67	-1.99	9.32	*0.203*	3.14	-1.57	7.85	*0.191*
*** immunotherapy***	-16.11	-31.60	-0.62	***0.042***	-9.26	-24.24	5.72	*0.225*	-11.14	-23.62	1.34	*0.080*
*** heparin***	-1.24	-6.82	4.33	*0.661*	-4.80	-10.19	0.59	*0.081*	2.76	-1.74	7.25	*0.228*
*** NIV***	1.14	-4.63	6.90	*0.699*	0.69	-4.88	6.27	*0.808*	-1.20	-5.85	3.44	*0.611*

*SF-36* the Short-Form 36-item questionnaire, *PCS* physical components score of the SF-36, *MCS* mental components score of the SF-36, *EQ-VAS* Visual Analogue Scale score of the EQ-5D, *CI* confidence interval, *NIV* noninvasive ventilation

**Table 4:** Association between alterations in BAI, BDI-II and PSQI, and higher scores in SF-36 and EQ-VAS, by univariable linear regression

	**PCS**				**MCS**				**EQ-VAS**			
	**beta**	**95%CI**		***p-*** **value**	**beta**	**95%CI**		***p-*** **value**	**beta**	**95%CI**		***p-*** **value**
BAI*	-0.84	-1.04	-0.65	** < ** ***0.001***	-0.93	-1.11	-0.75	** < ** ***0.001***	-0.59	-0.74	-0.44	** < ** ***0.001***
BDI-II*	-1.10	-1.32	-0.88	** < ** ***0.001***	-1.22	-1.42	-1.01	** < ** ***0.001***	-0.74	-0.91	-0.57	** < ** ***0.001***
PSQI*	-0.29	-0.50	-0.08	***0.007***	-0.22	-0.43	-0.01	***0.037***	-0.24	-0.39	-0.08	***0.004***

*PCS* physical components score of the SF-36, *MCS* mental components score of the Short-Form 36-item questionnaire, *EQ-VAS* Visual Analogue Scale score of the EQ-5D, *CI* confidence interval, *BAI* the Beck Anxiety Inventory, *BDI-II* the Beck Depression Inventory, *PSQI* the Pittsburgh Sleep Quality Index. *evaluated in a subgroup of 265 participants

## Discussion

Our study involved a large number of patients from Italy who were systematically assessed for neuropsychological signs and symptoms at three timepoints after COVID-19, including a high proportion of patients without previous hospitalization, followed up to more than one year after the acute infection. Patients reported a significantly worse perception of physical and mental health *status* compared to the Italian normative group, and it remained stable over time, even one year after the acute infection. Other Italian studies assessing HRQoL through the same instruments (SF-36 and EQ-5D), were mainly focused on previously hospitalized patients evaluated not more than six months after COVID-19 [27–31]. One of the key findings of our study was the evidence of a persistent poor health *status* perception in the post-COVID-19 period. Similarly to previous studies done using SF-36, the most affected domains and the least affected domain were physical role and physical function, respectively [32–34]. When compared to the Italian normative group, both SF-36 (PCS and MCS) and EQ-VAS scores were lower in the study population. Interestingly, these ratings remained stable over the period of observation and, for the mental component of SF-36 and for EQ-VAS, they were even lower at the last evaluations. These findings emphasize that the impact on quality of life in the post-COVID-19 period, is not restricted to the first few months after the acute infection, but it should be considered a concern also at distance. Here, we have analyzed only the first evaluation of the participants, performed during their first referral at our outpatient clinic, even when it occurred several months after the infection, highlighting the necessity of a prolonged follow up. Even though, in this study, details on signs and symptoms of post-COVID-19 syndrome were not reported, the persistence of a poor health *status* perception could be directly related to the persistence of such symptoms, providing further evidences of the considerable effect of this condition on quality of life.

A recent systematic review [6] reported that the most common factors associated with lower levels of HRQoL, were female sex, older age, the presence of co-morbidities and developing critical illness. Similarly, a previous structured review [7], focusing on the impact of both acute and long COVID-19 on HRQoL, found that age, gender, severity of illness, comorbidity, income and educational level of the patients, were factors related to a worse perception of HRQoL. Our results were partially consistent with these findings. If the association of lower scores in EQ-VAS, MCS and PCS with female gender was strong and clear, the correlation to disease severity was indirect. In fact, a worse perception of HRQoL was observed mainly among patients with comorbidities and patients treated, during the acute phase, with corticosteroids and, only for PCS, immunotherapy. These features may be considered an indirect sign of disease severity, even though the use of noninvasive ventilation was not related to lower scores. Moreover, gender-based inequalities in health and in quality of life, have been frequently documented in several settings [35–37], probably due to the different exposure and vulnerability to specific determinants of health [35], and this phenomenon was particularly evident in the context of COVID-19, considering that women seemed to suffer more frequently from long COVID symptoms [8, 38] with the consequent impact on the perception of health *status* and quality of life. As expected, considering the stable scores in SF-36 subscales during the entire period of observation, any association was found between HRQoL and distance from the acute phase. Finally, we did not observe any evidence of association with age, analyzed as a continuous variable, and we did not investigate income and educational level. Surprisingly, our study showed a better perception of MH in patients with previous hospitalization and it could be determined by the fact that patients managed at their own home, experienced the lockdown period and felt worried about the disease progression and potential complications, and insufficiently reassured about the symptoms they experienced. As a result, their perception of these symptoms could be exacerbated and their psychological sphere was possibly affected more the ones hospitalized. On the contrary, the hospitalization could offer more reassurance to the patients by inhibiting their anxious thoughts.

Regarding the presence of anxious-depressive symptoms and sleep disorders, we found that it was associated with a worse perception of health *status* and QoL. Such correlation is well documented also for other diseases, acute and chronic, and highlights the need to deal with neuropsychiatric symptoms during the post-COVID-19 period [39, 40].

Strengths of the present study were the prospective design and, as already underlined, the large sample size, including patients, with milder forms of COVID-19 and with a long follow up period, systematically evaluated at each visit; moreover, scales and questionnaires were face-to-face administered by health-professionals and neuropsychologists, decreasing the likelihood of misunderstanding and missing responses. The study had also some limitations. First of all, it was a single-center study without a control group of patients and with a cross-sectional design, thus the absence of longitudinal data could limit the generalizability of the results. Furthermore, for this analysis, we did not collect details on symptoms and we are aware of the potential selection bias due to the increased willingness of more symptomatic patients to take part in a follow-up study. Finally, the long-term impact of post-COVID-19 syndrome on self-reported dimensions of HRQoL was evaluated using only classical psychometric measures, not including any clinimetric assessment, which has been proposed as an innovative approach for assessing and measuring HRQoL in clinical settings [41, 42].

## Conclusions

The findings of the present analysis confirm the high impact of post-COVID-19 syndrome on the perception of health *status* and quality of life, when compared to the Italian reference group, even more than one year after the acute phase of infection and also among patients without a previous hospitalization. Factors as female gender, presence of comorbidities and disease severity, seem to characterize patients at a higher risk and may be considered a target population for focused interventions. Moreover, it is likely to assume that the health *status* perception is also affected by stress factors related to the pandemic isolation condition and to the infectious event itself. Indeed, COVID-19 should be seen as a traumatic event. Further large-scale prospective longitudinal studies focusing on the ability to respond to the traumatic event, especially among those patients who experience the infection, are needed to obtain a thorough knowledge of peri- and post-traumatic implications of the SARS-CoV-2 pandemic and of the seriousness of the psychological distress on the world population. Furthermore, future studies applying clinimetric indices are also needed to longitudinally assess the clinical impact of post-COVID-19 syndrome. Finally, a systematic monitoring of Patient Reported Outcomes and quality of life is recommended to properly manage patients in the post-COVID-19 period, for whom a multidimensional patient care must be ensured by the health care system, coordinated by a dedicated long COVID service with multidisciplinary support.

## Data Availability

The raw data generated and/or analyzed within the present study are available in our institutional repository (rawdata.inmi.it), subject to registration. In the event of a malfunction of the application, the request can be sent directly by e-mail to the Library (biblioteca@inmi.it). No charge for granting access to data is required.
